# Synchronic renal cell carcinoma associated with fibromixoid sarcoma: A rare finding

**DOI:** 10.1016/j.ijscr.2020.02.051

**Published:** 2020-02-28

**Authors:** Jose Genilson Alves Ribeiro, Ângelo Maurílio Fosse Junior, Victor Bastos Frade, Guilherme Gonçalves Rocha, Lucas Alves Sarmento Pires, Caio Fernando Cardoso Souza, Marcio Antonio Babinski

**Affiliations:** a Antonio Pedro University Hospital, Fluminense Federal University, Niterói, Rio de Janeiro, Brazil; b Morphology Department, Fluminense Federal University, Niterói, Rio de Janeiro, Brazil

**Keywords:** Renal cell carcinoma, Case report, Clear cell type, Nephrectomy, Tumor resection, Woman

## Abstract

**Introduction:**

Renal cell carcinoma comprises over 90% of renal cancers, thus, it is the most common form of renal neoplasia. This carcinoma can often present itself in a variable fashion, ranging from incidentalomas to metastatic diseases. Furthermore, the most common metastasis associated with this type of carcinoma occurs in the lungs, bones or liver. We aim to report a case of renal cell carcinoma which presented together with a fibromixoid sarcoma.

**Case presentation:**

A 50 year-old woman presented with hematuria, weight loss, asthenia and right lumbar pain that started 7 months prior to the consult. CT scan was performed and revealed a solid injury of 10 cm in the right kidney together with a mass in the left flank. Total right nephrectomy was promptly performed and the patient was submitted 3 months later to a tumoral resection of the abdominal wall. Histopathological findings revealed a primary renal cell carcinoma and the second, metastatic tumor was shown to be a fibromixoid sarcoma. The patient was not submitted to chemotherapy and is currently under follow-up with the surgery and oncology staffs, without showing any symptoms.

**Discussion:**

Renal cell carcinoma usually presents itself together with secondary tumors on the lungs and bones. The association of this type of carcinoma with a fibromixoid sarcoma of the abdominal wall is rare and poorly reported in the literature.

**Conclusion:**

This case reports shows a successful treatment regarding this rare association, which can help other physicians to re-evaluate their medical conduct.

## Highlights

• Renal cell carcinoma can be associated with other tumors.
• Its association with fibromixoid sarcoma is rare.
• We report a case of the aforementioned association.
• Nephrectomy and removal of the fibromixoid sarcoma was done.
• Patient was released after surgery with no complications.

## 1 Introduction

Renal cell carcinoma (RCC) comprises over 90% of renal cancers, thus, it is the most common form of renal neoplasia. Furthermore, its prevalence among the general population is increasing due to better diagnostics exams, which allows early identification, as such, mortality rates of RCC are diminishing over the decades [1,2].

RCC can often present itself in a variable fashion, ranging from incidentalomas to metastatic diseases. Moreover, at least one third of the RCC are known to be metastatic, although the prevalence of incidentalomas is rising [3].

RCC can present itself with other forms of primary tumors, albeit this is a very uncommon finding. RCC has been described together with stomach and pulmonary cancers, although the association of RCC with prostate, pharynx and esophageal malignant tumors has been reported in the literature [4].

Fibromixoid sarcoma is a malignant and misleading tumor, as its histological features are seemingly bland. These tumors usually arise in the deep soft tissues of the proximal extremities or trunk of young adults. They have a predilection for young adults, however, these tumors can be found in individuals between 10 and 69 years-old, according to the literature [5,6].

The work presented herein aims to report a rare case of RCC associated with a fibromixoid sarcoma in a female patient and to discuss this rare finding. Furthermore, this case has been reported in line with the SCARE criteria [7].

## 2 Case report

**Fig. 1:**
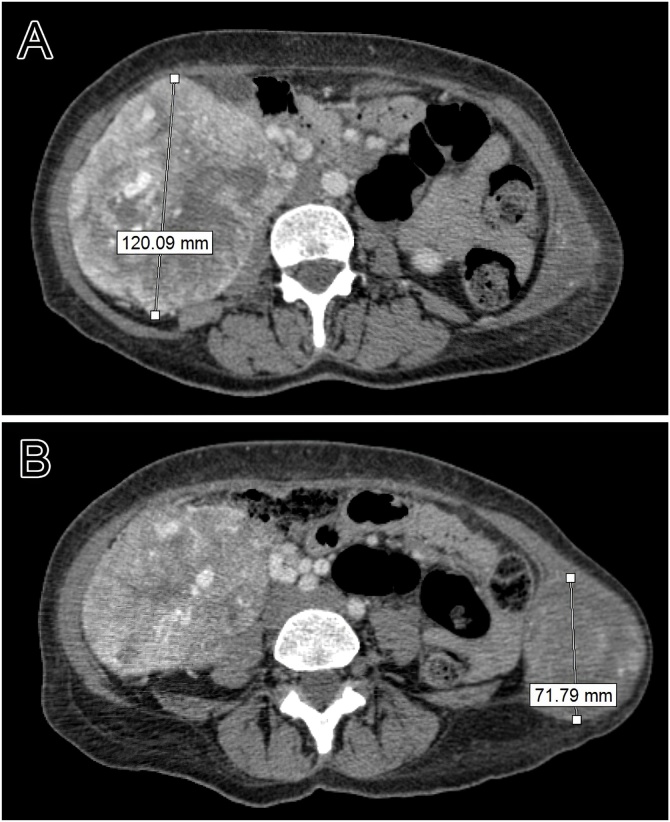
Contrast abdominal CT revealing mass in the right kidney (A) and a mass in the left abdominal wall (B).

A 50-year-old female patient presented with hematuria, massive weight loss (45 kg), asthenia and right lumbar pain 7 months prior to the consult. Previous blood work-up revealed anemia and previous CT and ultrasound revealed a solid injury of 10 cm on the right kidney and a mass in the right flank (Fig. 1). The patient denied previous family history of neoplasia and previous diseases, although she confirmed smoking habits.

Physical exam revealed a palpable mass with roughly 10 cm at the right hypochondrium and another palpable mass situated at the dorsal surface of the left lumbar region with roughly 5 cm. The patient had regular and stable vital signs.

Routine admission blood work-up confirmed anemia, and a contrasted CT scan revealed a solid mass on the right kidney (12 cm) and a solid mass situated on the muscle plane of the abdominal wall muscles (7 cm). The patient was promptly submitted to a right total nephrectomy, retroperitoneal lymphadenectomy, right total adrenalectomy and a cavotomy with the purpose of removing the caval thrombus.

**Fig. 2:**
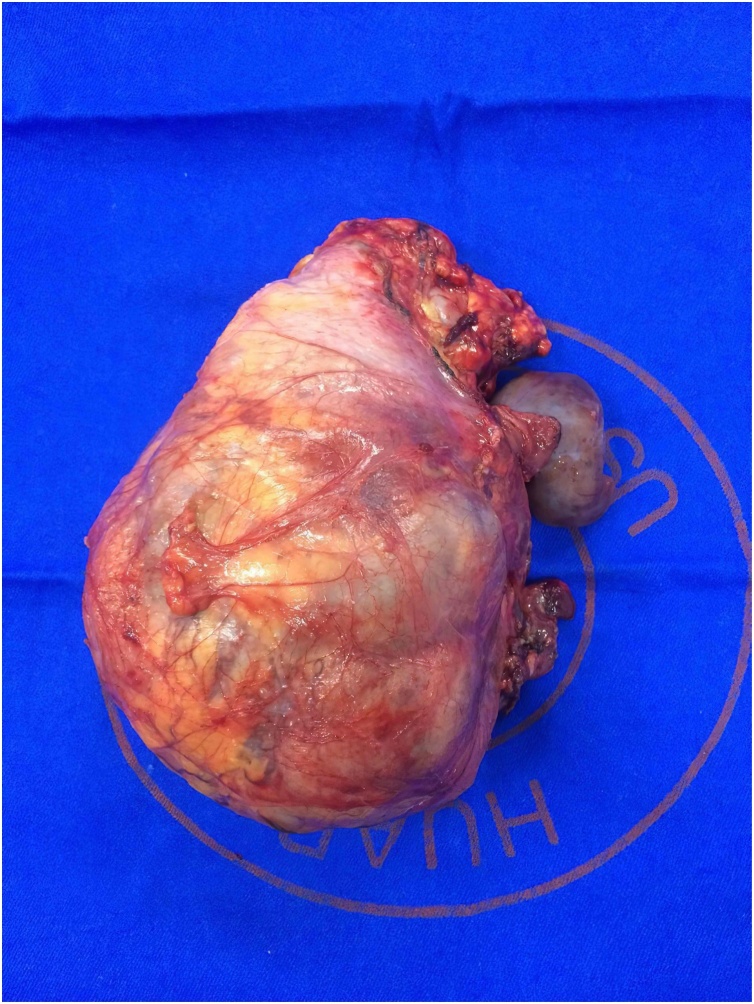
Right kidney. The tumor (10 × 8,5 × 7 cm) occupies almost all of the kidney, invading parenchyma and the renal pelvis.

**Fig. 3:**
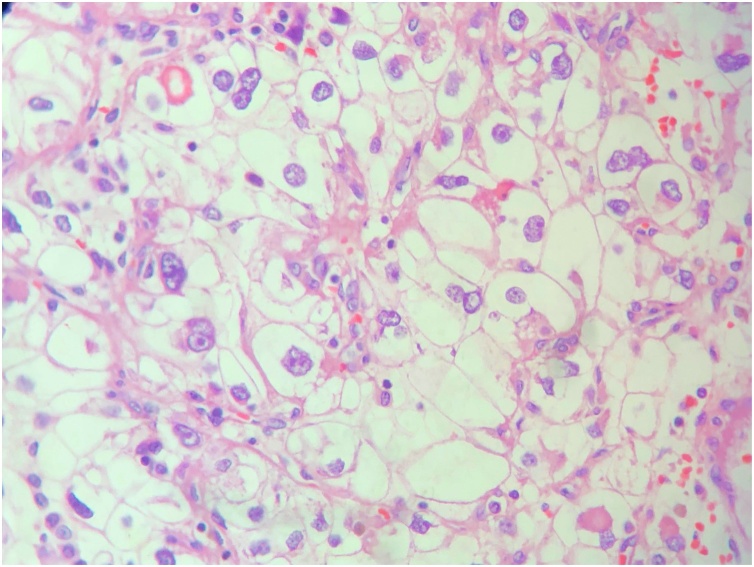
The renal clear cell carcinoma is shown. An alveolar architecture of cells with clear cytoplasm (from lipid/glycogen) can be seen. Hematoxylin/Eosin, X400.

The tumor and the excised kidney (Fig. 2) were submitted to a histopathological analysis, which confirmed renal cell carcinoma of clear cell type with sarcomatoid cell type component (Fig. 3). The histologic grade of the tumor was 4 and the tumor was associated with neoplastic thrombus in the inferior vena cava (pT3b).

The surgery was uneventful and since the patient made a good recovery from the procedure, she was subsequently discharged from the hospital. One month later, the patient returned to the hospital for a tumor resection of the abdominal wall, which raised suspicion of metastasis.

**Fig. 4:**
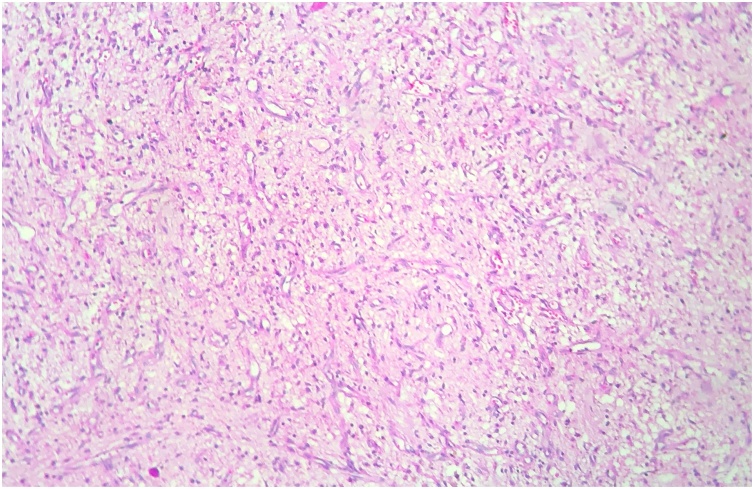
The fibromixoid sarcoma is shown. Myxoid areas and a swirling whorled growth pattern can be seen. Hematoxylin/Eosin, X100.

After resection, the tumor was sent to histopathological analysis, which revealed a neoplasia with myxoid component and a predominance of fusiform cells (Fig. 4). The immunohistochemical profile was unspecific, with characteristics of low grade fibromyxoid sarcoma.

Since the surgery was successful and there were no complications during post-op, the patient was discharged and subsequently monitored during routine check-ups.

## 3 Discussion

Despite the presentation of both tumors, it is not possible to confirm an association between them. The case presented herein can correspond to either metastasis to subcutaneous tissue and skeletal muscle or to multiple primary tumors (including benign tumors, such as lipoma and hemangioma, and malignant tumors). Although not included among the most common sites of metastasis, a study showed that 0.4% of the cases of RCC had metastases in muscular or skeletal tissues [8].

Malignant tumors synchronous to RCC are rarer. Although there were reports in the literature of synchronous sarcomas due to RCC, these reports did not mentioned the probable histological type presented herein [9].

For all cancers, primary multiple tumors have a frequency ranging from 2 to 17%. This definition includes metachronous tumors (multiple primary tumors developing at intervals of at least six months) and synchronous (two or more tumors identified simultaneously in the same patient or a second tumor identified up to six months after the initial diagnosis) [10].

A study showed that multiple primary tumors including urinary tract neoplasias may reach 9% of all urologic cancers, with bladder and prostate being the sites most associated with this type of manifestation [11]. However, no epidemiological series have been published recently to confirm this statement.

The pathogenesis of the RCC is not completely understood. Risk factors such as age (between sixth and seventh decade of life), obesity and smoking have been recognized [1] and were all present in the reported case. This group of neoplasms is heterogenous and includes cancers with different genetic and molecular changes behind several documented histological subtypes. Among them, the most frequent are clear cells (corresponding to most cases, including the one reported herein), papilliferous and chromophobic [12].

Diagnosis is based on clinical findings and imaging exams. Signs and symptoms include hematuria, flank pain, palpable abdominal mass, all of which have negative prognostic implications. Systemic symptoms may be due to metastases or paraneoplastic syndromes, including hypertension, fever, consumption syndrome, anemia, hypercalcemia and others [13].

However, with the increase in use of imaging exams for other pathologies, many diagnoses of renal neoplasia are being made accidentally during scans, as such, only 30% of patients are diagnosed based on the symptoms [12]. Ultrasonography often detects the tumor, but a CT scan or magnetic resonance imaging is usually needed to confirm the diagnosis. The image allows one to characterize the mass, to identify the extent of the tumor, the presence of metastasis and venous involvement, which is important for a staging of the disease [12]. In addition to conventional imaging exams, technologies such as texture analysis and functional imaging (diffusion and perfusion) have shown promise in differentiating types of renal tumors [14].

With the emergence of small masses discovered accidentally, many conservative therapies have been more widely used, such as active surveillance, minimally invasive techniques such as cryotherapy and radiofrequency ablation, and partial nephrectomies [12]. However, if renal parenchyma cannot be spared, radical nephrectomy should be the procedure of choice, especially for tumors larger than 7 cm and with local invasion characteristics. Typically in these cases, resection of adjacent blunt organs and thrombectomy are also performed. This strategy can cure up to 60% of patients [13].

Despite that, the treatment of metastatic diseases - such as the one presented herein - hardly possesses a full recovery proposal. Radical nephrectomy (cytoreductive surgery) seems to benefit many patients with metastatic disease; although, it should not be used indiscriminately in any metastatic patient. This type of surgery is indicated for massive lesions (which comprises over 75% of the renal parenchyma) without metastases to the central nervous system and liver and patient with good status and good cardiac and pulmonary function [13]. Our conduct, therefore, was adequate in this case and is corroborated by the literature.

Another strategy that is used in metastatic RCC therapy is systemic therapy. Historically, the therapy was based on interleukin 2 and gamma interferon, but the outcome was poor and had many adverse effects. Recently, new targets for systemic therapy have been studied, and the most commonly used agents are VEGF receptor (vascular endothelial growth factor) inhibitors and mTOR inhibitors. However, the prognosis of the patients who undergo this type of therapy is still unsatisfactory, with most of them finally evolving to death due to complications of the disease [12,15].

On the other hand, low-grade fibromyxoid sarcoma present in this report is much rarer than RCC, corresponding to 0.18 per million individuals, and affects mainly young adults. Most cases present a painless deep mass in the proximal region of limbs and trunk [5]. Its treatment is generally similar to that of other low-grade sarcomas, with wide excision and possible adjuvant radiotherapy [16].

As for the synchronic tumors in general, the conduct can be challenging and varies according to the histological types, the staging of the disease and the prognosis of the patient. Cases such as ours must be discussed in a multidisciplinary setting, as a consensus regarding which therapeutic strategy may require more than one team [10].

In our case, the most important disease responsible for a poor prognosis was undoubtedly the metastatic RCC. The conduct of performing a radical nephrectomy allied to subsequent chemotherapy was based on the literature, as previously stated. As for the fibromixoid sarcoma, the excision procedure with surgical margin is also recommended in general.

## 4 Conclusion

The present work reported an association between RCC and fibromynoid sarcoma, which is a rare finding and poorly documented in the literature. Despite that, it is impossible to say whether this manifestation of synchronic tumors was coincidental or if there was some genetic or environmental connection between the two cancers.

However, it is possible that such synchronic tumors are underdiagnosed, since not all masses in a metastatic RCC are histologically analyzed, as was the case. We believe that our case adds data to the literature regarding the association of both aforementioned tumors.

## Sources of funding

There were no funding for this work.

## Ethical approval

The case report in question is exempt from ethical approval from the institution, as the patient already signed a consent form.

## Consent

Written informed consent was obtained from the patient for publication of this case report and accompanying images. Furthermore, no personal information regarding the patient is present in this study.

## Author contribution

Admission, diagnosis and treatment: Ribeiro, Fosse Junior, Frade, Rocha.

Getting consent from the patient: Fosse Junior, Babinski.

Data analysis: Frade, Rocha, Babinski.

Review of the literature: Ribeiro, Frade, Rocha, Pires.

Writing of the paper: Pires, Babinski.

English translation: Pires/Babinski.

## Registration of research studies

The study was performed according to the Declaration of Helsinki 2013 and the SCARE Guidelines.

## Guarantor

I, Marcio Antonio Babinski accept full responsibility for the work and the conduct of the study, had access to the data, and controlled the decision to publish.

## Declaration of Competing Interest

There are no conflicts of interest.
